# Supplementary material for: ADAM10 Plasma and CSF Levels Are Increased in Mild Alzheimer’s Disease
Source: Int J Mol Sci. 2021 Feb 28;22(5):2416. doi: 10.3390/ijms22052416 (PMC7957802; doi:10.3390/ijms22052416)
Supplement: Supplementary file 1 [file ijms-22-02416-s001.pdf]

ADAM10 Plasma and CSF Levels Are Increased in Mild Alzheimer's Disease

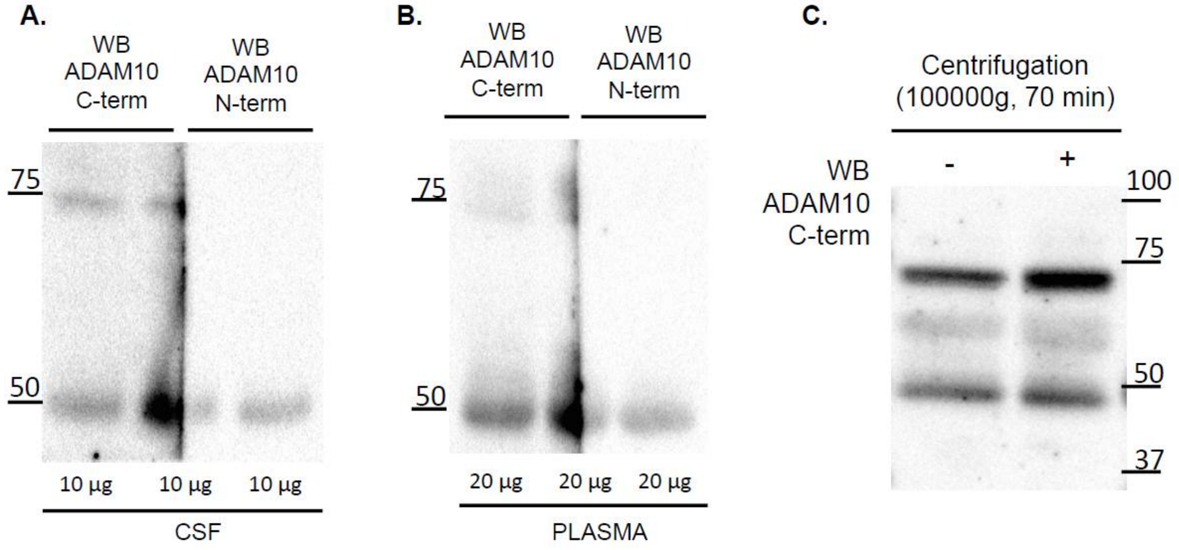

Figure S1. The 50 kDa ADAM10 form has both N-and C-terminal domain and is not associated to extracellular vesicles. The 50 kDa ADAM10 form detected in CSF and plasma is recognized by both ADAM10 antibodies raised against ADAM10 C- and N-terminal domain. Three aliquots of the same samples of CSF (A) and of plasma (B) were loaded onto a SDS-PAGE. The central lane was cut in the middle to incubate each half with either ADAM10 N-terminal antibody or the ADAM10 antibody raised against the C-terminal region. Both antibodies recognize the band at 50 kDa, while a band at 75 kDa is recognized only by the ADAM10 C-terminal antibody. (C) An aliquot of CSF and an aliquot of the supernatant collected after CSF centrifugation (100000 g, 70 min), to remove extracellular vesicles, were loaded onto SDS-PAGE and Western Blot analysis performed with the antibody recognizing ADAM10 C-terminal region. The centrifugation doesn't affect the detection of ADAM10 in CSF.
